# Assessment of the Efficacy, Safety, and Effectiveness of Weight Control and Obesity Management Mobile Health Interventions: Systematic Review

**DOI:** 10.2196/12612

**Published:** 2019-10-25

**Authors:** Elisa Puigdomenech Puig, Noemí Robles, Francesc Saigí-Rubió, Alberto Zamora, Montse Moharra, Guillermo Paluzie, Mariona Balfegó, Guillem Cuatrecasas Cambra, Pilar Garcia-Lorda, Carme Carrion

**Affiliations:** 1 Agència de Qualitat i Avaluació Sanitàries de Catalunya Barcelona Spain; 2 Red de Investigación en Servicios de Salud en Enfermedades Crónicas Barcelona Spain; 3 eHealth Lab Barcelona Spain; 4 eHealth Center Universitat Oberta de Catalunya Barcelona Spain; 5 Faculty of Health Sciences Universitat Oberta de Catalunya Barcelona Spain; 6 Interdisciplinary Research Group on ICTs Barcelona Spain; 7 Corporació de Salut del Maresme i la Selva, Hospital de Blanes Blanes Spain; 8 Grup de Medicina Traslacional i Ciències de la Decisió Departament de Ciències Mèdiques, Facultat de Medicina Universitat de Girona Girona Spain; 9 CIBER Epidemiología y Salud Pública Barcelona Spain; 10 Clínica Sagrada Família CPEN SL Servei d'Endocrinologia i Nutrició Barcelona Spain

**Keywords:** mHealth, obesity, overweight, systematic review, technology assessment

## Abstract

**Background:**

The use of apps to tackle overweight and obesity by tracking physical and dietary patterns and providing recommendations and motivation strategies to achieve personalized goals has increased over recent years. However, evidence of the efficacy, effectiveness, and safety of these apps is severely lacking.

**Objective:**

The aim of this study was to identify efficacy, safety, and effectiveness criteria used to assess weight control, overweight, and obesity management in mobile health (mHealth) interventions through a systematic review.

**Methods:**

PubMed, PsycINFO, Scopus, UK Trial Database, ClinicalTrials.gov, and the Cochrane Library were surveyed up to May 2018. All types of clinical studies were considered. A total of 2 independent reviewers assessed quality using Scottish Intercollegiate Guidelines Network (SIGN) criteria. Ratings were used to provide an overall score for each study (low, moderate, or high). Data were synthesized in evidence tables.

**Results:**

From 233 potentially relevant publications, only 28 studies were included. Of these, 13 (46%) were randomized control trials, 11 were single-arm studies (39%), 3 were nonrandomized controlled trials (11%), and 1 study was a cluster randomized trial (4%). The studies were classified as low (15), high (7), and moderate (6) quality according to SIGN criteria. All studies focused on efficacy, with only 1 trial mentioning safety and another 1 effectiveness. In 11 studies, the apps were used as stand-alone interventions, the others were multicomponent studies that included other tools for support such as sensors or websites. The main management tool included in the apps was feedback messaging (24), followed by goal-setting mechanisms (20) and self-monitoring (19). The majority of studies took weight or body mass index loss as the main outcome (22) followed by changes in physical activity (14) and diet (12). Regarding outputs, usability, adherence, and engagement (17) were the most reported, followed by satisfaction (7) and acceptability (4).

**Conclusions:**

There is a remarkable heterogeneity among these studies and the majority have methodological limitations that leave considerable room for improvement. Further research is required to identify all relevant criteria for assessing the efficacy of mHealth interventions in the management of overweight and obesity.

**Trial Registration:**

PROSPERO CRD42017056761; https://tinyurl.com/y2zhxtjx

## Introduction

### Background

Obesity and overweight are considered major public health concerns because of their high prevalence and association with various health complications including cardiovascular disease, type 2 diabetes, and cancer [1,2]. As the aspects that influence overweight and obesity are diverse—comprising individual, genetic, and environmental factors—their prevention and treatment are also complex. For a successful treatment, multifactorial approaches are required, with diet and exercise plans reinforced with psychological therapy and behavioral change strategies [3].

In recent years, we have witnessed a revolution in the use of apps within personal health care, as they are fast, flexible, handy, versatile, manageable, and illustrative tools that can empower patients. Hence, mobile health (mHealth) can play an important adjuvant role in the prevention and treatment of overweight and obesity by tracking physical activity (PA), enabling self-reporting of dietary patterns, providing recommendations to achieve healthier habits, guidance, advice, tips, and motivational strategies to achieve personalized goals; all are relevant aspects for the prevention and treatment of obesity, as recognized in numerous guidelines [3,4].

The Global Observatory for eHealth of the World Health Organization (WHO) defines mHealth as “medical and public health practice supported by mobile devices, such as mobile phones, patient monitoring devices, personal digital assistants, and other wireless devices” [5]. The management—and in some cases the prevention—of chronic diseases has been one focus in recent developments in both electronic health and mobile health (eHealth and mHealth) [6]. There are over 325,000 health apps on the market, with the most downloaded being those relating to PA and weight control: that is, those that support a healthy lifestyle [7]. However, information on how the effectiveness, efficacy, and safety of mHealth apps in overweight and obesity management are assessed is severely lacking. It is important to note that according to mHealth publishers, over 53% of their health apps portfolio available in 2015 were downloaded less than 5000 times [7]. Evidence of the efficacy of mHealth app strategies in improving chronic health and well-being is mixed; therefore, while some mHealth interventions show promise in supporting weight management [8,9], others do not [10,11]. Numerous efforts to address this challenging issue are underway and some aspects that may be linked to a lack of efficacy have been identified. These are, among others, the poor quality of many apps, a lack of guidance on the usefulness of an app, and a low level of support from health professionals [12,13]. Should mHealth apps be rigorously evaluated to ensure they provide evidence-based effectiveness, safety, and efficacy? Up to now, mHealth evaluation methodology has not deviated from customary methods (mainly randomized controlled trials [RCTs]), despite claims that alternative, shorter, and more inexpensive design methods are required [14].

There are several initiatives attempting to define how apps should be evaluated. However, all of these consider only partial aspects of evaluation [15]. Although medical regulatory bodies have not validated the safety and quality of these technologies, individuals have adopted mHealth devices as self-management aids. However, medical professionals are often at a loss as to how to relate to them [16]. Owing to this rapid consumer-based introduction to the world of patient health aids, mHealth solutions present unique and stakeholder-specific challenges to the medical environment. Patients, health care providers, administrators, authorities, and mHealth developers alike are operating without a clear direction, which may lead to problems, including the improper use of mHealth interventions by individuals and the inability of medical systems to react due to a lack of technological and organizational support. Users and health care professionals should be aware of the quality of health apps they use or prescribe. The use of classic methodologies such as RCTs may not be the optimal procedure for evaluating all the dimensions of mHealth. Ideally, clinicians, health administrations, and users need instruments that enable the evaluation of e-interventions as a whole. From a global perspective, these instruments should facilitate the process of verification, validation, impact assessment, and certification that ought to be a requirement for all mHealth implementation.

This lack of rigorous evaluation is an increasing concern for health authorities. A number of recommendations to ensure a minimum quality of mHealth interventions have already been defined by the WHO Technical Evidence Review Group [17]. In addition, both the *Food and Drug Administration* [18] and the *European Commission* [19] have made several attempts to establish mHealth assessment and, where appropriate, certification criteria. However, in such a continuously evolving field, it has been difficult to reach a consensus.

### Objectives

The aim of this paper was to undertake a systematic review of efficacy, safety, and effectiveness assessment criteria in use, including both outputs and outcomes, to assess weight control, overweight, and obesity management in mHealth interventions. These criteria will later be included in a tool for assessing mHealth interventions intended to manage overweight and obesity.

## Methods

This systematic review was prospectively registered with PROSPERO (CRD42017056761) [20]. The Preferred Reporting Items for Systematic Reviews and Meta-Analyses (PRISMA) statement was used as a guide for reporting this review [21]. Owing to the methodological and statistical heterogeneity of the included studies, a descriptive approach was adopted in the research synthesis.

### Eligibility Criteria

Any trial that assessed the efficacy and/or safety and/or effectiveness of mHealth-based interventions for overweight or obesity management was considered. No restrictions in terms of target population were foreseen. We define efficacy as changes in lifestyles on the basis of diet and PA in a controlled population; effectiveness in the general population; and safety as a lack of adverse effects resulting from mHealth interventions. Studies carried out with less than 10 individuals were excluded. We assessed the quality of trials according to the Scottish Intercollegiate Guidelines Network (SIGN) criteria [22]. Taking the objective of this review into consideration, all studies were included regardless of quality.

### Information Sources

A systematic search was conducted in the following databases: MEDLINE, EMBASE, PsycINFO, The Cochrane Library (Cochrane Database of Systematic Reviews, Cochrane Central Register of Controlled Trials [CENTRAL]), UK Trial Database, and Scopus. This survey was supplemented through the snowballing technique to identify relevant articles in the references of those returned by the search. A manual search was also conducted on the indices of the following publications: *Journal of Medical Internet Research* and *JMIR mHealth and uHealth*. The survey period included all articles published up to May 2018. All types of clinical studies published in English, French, or Spanish were considered.

### Search Strategy

The search strategy included both controlled vocabulary and free-text terms. The terms used were apps, mHealth, eHealth, overweight, obesity, efficacy, security, safety, effectiveness, and evaluation (see Multimedia Appendix 1).

### Study Selection and Data Collection Process

All identified references were imported into Mendeley v1.18 (Elsevier) and duplicates eliminated. A total of 6 researchers undertook the review process, which was conducted in 2 stages. First, each article identified was randomly assigned to 2 reviewers to independently review the title and abstract. Articles that met the inclusion criteria were full-text reviewed and quality-assessed by 2 independent reviewers. In cases of disagreement, a third reviewer made the final decision. Study features and outcomes were entered into a database specifically designed for this review. Risk of bias was assessed according to SIGN codes for study assessment [22]. Those trials that were clearly of an adequate quality were graded as *high or ++* (very low risk of bias) or *moderate or +* (low risk of bias), while those of insufficient quality were graded as *low or–*(high risk of bias).

## Results

### Selection of Studies

**Figure 1:**
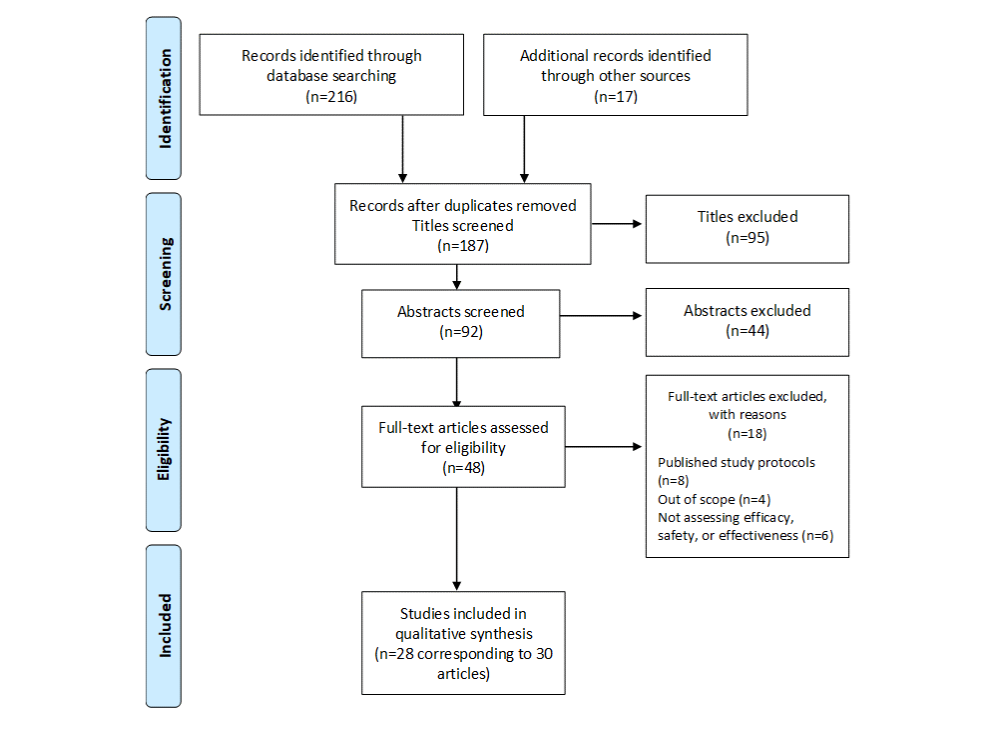
Preferred Reporting Items for Systematic Reviews and Meta-Analysis (PRISMA) flow diagram of selection of papers for inclusion in the review.

A total of 233 potentially relevant publications (17 from a manual search) were identified as eligible. From these, 19.7% (46/233) were identified as duplicates. From the remaining (187), only 49.2% (92/187) were accepted for abstract review. Out of these, 47% (44/92) were excluded for not following inclusion criteria. A full-text review was conducted on 48 studies. After peer review, 30 articles corresponding to 28 different studies (62.5% from the total included for full-text review) were finally included in this nonquantitative review. The exclusion criteria were as follows: published study protocols (n=8), out of scope studies that were not using an mHealth intervention (n=4), or those studies in which final outcomes were other than efficacy or safety (n=6; Figure 1; see Multimedia Appendix 2).

The main characteristics of the 28 studies included are detailed in Multimedia Appendix 3. Studies appear in alphabetical order of the first author within chronological years. All selected studies focus on efficacy; only 1 of them assesses effectiveness and 1 also focused on safety, although this was not the main outcome of the study.

In total, 46% of the studies (13/28) are RCTs, 1 is a cluster-randomized trial [23], 3 are nonrandomized controlled trials, and the remainder (11) single-arm trials; 2 of the RCTs include more than 1 intervention. Carter et al [24] studied the efficacy of a smartphone app or website in self-monitored weight management, and Hurkmans et al [25] compared 1 stand-alone app intervention with face-to-face and blended interventions. All studies compare pre and post outcomes to analyze the intervention’s efficacy. According to SIGN criteria, the majority of studies are of low (15) or moderate (6) quality, with only 7 studies reaching high quality. A low quality rating most often resulted from small sample size, inadequate length of study, or possible selection and information bias.

The number of participants ranged from 10 to 1012, but most studies (17) covered less than 100 people. One trial [26] had 15,310 participants, but the majority (83%) remained nonactive during the intervention. Most studies had a majority of adult women; in 6, all participants were women. There are also 4 studies targeted at children and teens. Most trials were targeted at people with overweight or obesity but no other health condition: exceptions targeted people with a severe mental illness [27], heart disease [28], type 2 diabetes [29] or prediabetes [30,28], cancer survivors [31,32], and people with metabolic syndrome [33].

Apart from one 24-month trial [34], the studies were conducted over short periods of time, ranging from 3 weeks to 6 months. The countries where the studies were carried out were the United States (17), Australia (3), Korea (2), the United Kingdom, Belgium, Spain, The Netherlands, China, and Israel.

### Elements Included in the Mobile Health Interventions

In regard to the specificities of mHealth interventions (Multimedia Appendix 4), only 39% (10 out of 28) focused on a specific stand-alone app, with the majority addressing multicomponent interventions—including armband sensors, pedometers, wireless scales, and other monitoring devices, or websites—for weight management, intended to increase PA, reduce sedentary habits, and/or improve dietary patterns. The most common elements included in the trials were the receiving of feedback messages (24 studies out of 28, 85%), setting of goals (20), and self-monitoring (19). These feedback messages could be personalized reminders, recommendations based on the self-monitoring, standard counseling or health coach counseling through the app, and/or a more synchronic intervention. New elements have been introduced to mHealth interventions in recent years, such as gamification [35,30,23,29,26], entertainment aspects [30,36,37,23,38], and peer contact through community blogs [39] or virtual teams [30] on social networks [37,27,40] such as Facebook [27,40,25] and We Chat [26]. It is worth mentioning that only specific frameworks were mentioned when defining strategies for behavioral change, such as the transtheoretical model of behavior change for TXT2BFiT [39] and CITY [34], intervention and Control Systems Theory for eBalance App [41], self-regulation theory for Balance It intervention [23], and increasing adherence, such as Mechanics-Dynamics-Aesthetics for With U App [40]. Social Cognitive Theory is the one most often referred to by the Vegethon app [42], Loose It app [31], and Alive-PD [30] for which several other frameworks were also considered: behavioral economics, positive psychology, and the theory of planned behavior. One study was based on the Diabetes Prevention Program [43] and LookAHEAD (Action for Health in Diabetes) trials [44]. One study was based on an addiction treatment approach [37].

### Output Tools and Measures

Although their main aim was to measure the efficacy of mHealth interventions, most of the selected studies also measured other outputs that might be relevant to determine primary outcome measures (23 out of 28, 82%). Multimedia Appendix 5 [45,46-50] shows the outputs and the main tools used to measure them.

#### Acceptability

A total of 4 studies out of 28 (14%) attempted to measure participants’ acceptance of the intervention, using mixed methods (survey, focus groups, and data performance tracking) [24,36,38,32]. Results showed that participants are willing to participate in these interventions, although receiving a smartphone [24] or doing it on a voluntary basis are elements that should be considered [32].

#### Usability/Adherence/Engagement

These 3 dimensions have been considered together, as the main analysis strategies used (data tracking and surveys) integrate all 3 aspects. Only 1 study used a validated questionnaire to assess usability [40], the System Usability Scale questionnaire. Several studies measured these outputs through different strategies, mainly data tracking. Results were very heterogeneous and depend on the study design and the specificities of each intervention.

#### Satisfaction

Only 7 studies analyzed the satisfaction rate of users [51,33,37,41,52,40,53], with 3 of these using standardized validated tools [54-56]. Results showed that most of the participants were very satisfied with the intervention, although a few considered the app too tedious to use.

#### Motivation to Lose Weight and to Continue the Intervention

Few studies [57,40] addressed continued motivation to lose weight after the intervention [41]. Only 1 of these [40] used a previously validated methodology, whereas the other 2 studies assessed motivation or intention to continue through a Likert scale [57] and self-reported questionnaires [41]. Results showed increases in users’ motivations and in the adoption of a positive attitude toward managing their overweight or obesity.

#### Perceived Peer Support

Out of the 7 studies dealing with peer support, only 2 attempted to assess the perception of this support [27,32]. Both showed a high perceived importance of peer support in reducing stress associated with the intervention.

### Outcome Tools and Measures

The end point outcomes of the selected studies were as follows: reduction of weight and body mass index (BMI) as well as fat mass and waist and hip circumferences; changes in dietary habits, PA, and screen time patterns; biochemical measurements; and blood pressure (Multimedia Appendix 6 [58-73]).

#### Weight and Body Mass Index

Most of the studies (22/28, 78%) considered reduction of weight and/or BMI as the main outcome with which to assess intervention efficacy. Devices used to measure weight and/or height were detailed in 17 trials, and only a few relied on self-reported data [74,26,53]. Partridge et al [74] did not report any differences between self-reported data and scale measures. All trials measured reduction in body weight, but in 3 studies [34,75,26] there were no differences between control and intervention groups; 3 other studies [35,41,25] noted differences between groups, but if statistical significance is taken as *P*<.05, these did not reach the threshold. Interventions that included face-to-face elements produced significantly better final outcomes [34,25]; 5 other 2-arm trials showed a clear and statistically significant reduction in body weight [24,76,74,53]. All pretest-posttest single-arm studies also measured weight reduction after intervention, but this was not always significant [38,52,32]; in one of these studies, considered to be of low quality, all weight was fully regained by 24 weeks after the intervention [52].

#### Fat Mass

Fat mass reduction was measured in 3 studies [35,33,40] through bioelectrical impedance and producing controversial results. In the 2 RCTs [35,33], fat reduction was statistically significant when comparing the control and intervention groups. In a single-arm trial [40], reduction was not statistically significant.

#### Waist and Hip Circumferences

Fukuoka et al [76] measured changes in hip circumference, noting significant changes in the intervention group. In total, 8 trials [30,31,41,38,40,77,26,25] measured changes in waist circumference, although the protocols in use varied or were not clearly specified. Results were controversial. Safran et al [41] and He et al [26] reported no changes, whereas 5 trials [30,31,38,40,77] identified a clear and significant reduction in waist circumference, whereas Hurkmans et al [25] recorded nonsignificant reductions.

#### Dietary Pattern

We identified 12 trials that assessed changes in dietary patterns [78,76,42,25,31-39,41-34,32,79]. All trials employed 2-arm pretest-posttest analysis, except for Quinitliani et al [32] and McCarroll et al [31]. Only 3 [31,33,23] did not use validated and previously published tests or questionnaires. A total of 6 studies [31-39,23,34,79] found no change when comparing fruit and vegetable consumption or the macronutrient composition of daily diet between 2 groups, although the intervention group appeared to adhere more closely to a Mediterranean diet [79] or were more likely to consume vegetables [39]. Other studies were able to demonstrate a clear improvement in dietary patterns: Fukuoka et al [76] observed a clear decrease in the intake of saturated fat; Mumah et al [42] identified a higher intake of vegetables; Safran et al [41] observed an improvement in diet quality; and Hurkmans et al [25] noted a clear and significant decrease in total energy intake. Both Nollen et al [78] and Quintiliani et al [32] perceived a statistically insignificant increase in fruit and vegetable consumption. In regard to sugar-sweetened beverages, 2 studies were able to measure a significant [76,39] or slight decrease [78]. Unexpectedly, participants in the single-arm study by Quintiliani et al [32] consumed more sugar-sweetened beverages after the intervention.

#### Physical Activity Pattern

In total, 14 of the 28 studies (50%) had PA pattern as a main end point. Various strategies were used to measure PA: (1) data tracking through accelerometers [36,79,25], pedometers [76], armband sensors [57,36], or logs from the apps [31]; (2) standard questionnaires such as International Physical Activity Questionnaire (IPAQ) or IPAQ-Short Form (IPAQ-SF) [33-74,32], the Paffenbarger Physical Activity Questionnaire [34], or a modification of the IPAQ questionnaire [41]; (3) semistructured interviews [79]; and (4) ad hoc questionnaires [23]. The most common measurements were daily number of steps [36-28,79,25] and time spent doing PA [57,36,31,74,41,27]. The number of metabolic equivalents of task [33,32] and weekly self-reported spent kilocalories [34] were also used.

All studies except 4 [33,23,34,79] showed an improvement in PA patterns. However, only 5 of these stated that the improvement was statistically significant [57,76,31,74,41].

#### Emotional Well-Being

As the intervention assessed was based on an addiction treatment approach, Pretlow et al [37] analyzed changes in self-esteem and the likelihood of turning to food when feeling stressed. They reported a significant improvement in self-esteem and control of participants’ eating. The McCarroll trial [31] analyzed changes in quality of life for cancer survivors. There were no differences before and after the mHealth intervention.

#### Screen Time

Nollen et al [78] studied possible changes in screen time but recorded no differences between the control and intervention groups.

#### Biochemical Measurements

As blood fasting lipids and glucose levels are usually high among people with overweight and obesity; 5 studies included these as secondary outcomes. Only Block et al [30] could report a significant improvement in triglyceride/high-density lipoprotein ratio; 2 studies [79,25] showed a trend toward reduction but the results were not significant. The other 2 trials did not measure any change in either fasting lipids or glucose [76,33].

#### Blood Pressure

Fukuoka [76], Willey [77], and Mao [53] tracked changes in blood pressure as a secondary outcome. The 3 trials were able to measure significant reductions in both systolic blood pressure and diastolic blood pressure.

#### Safety

One high-quality trial [33] considered safety as an outcome to be measured. The aim of this study was to evaluate the effect of SmartCare intervention in patients with metabolic syndrome. They identified a number of mildly adverse events (14.2% in the intervention group and 13.3% in the control group). There were also serious adverse events: 1.4% corresponding to 3 cases in the intervention group, including 1 ankle fracture; and 2.4% (5 cases) in the control group, including dislocated vertebra, stress urinary incontinence, and the need for a knee operation.

#### Effectiveness

Only 1 study was targeted at the general population. He et al [26] conducted a low-quality trial on 15,310 people. No differences between the intervention and control group were shown in terms of losing weight.

## Discussion

### Principal Findings

In this systematic review, we have identified the range of dimensions and tools used to assess the efficacy of mHealth interventions intended to manage overweight and obesity. We have provided a descriptive analysis of 28 clinical trials along with an account of the components and elements included in each intervention. Outputs and outcomes used for the evaluation of trials have also been identified. No specific criteria for assessing safety or effectiveness have been identified due to the small number of studies focused on these aspects.

Our results show that researchers use the following primary end points to measure a study’s success: (1) reduction in weight and/or BMI; (2) reduction in fat mass; (3) reduction in waist and hip circumference; (4) improvement in dietary habits/patterns; (5) increase in PA; (6) increase in emotional well-being; (7) decrease in screen time patterns; (8) improvement in biochemical measures; and (9) decrease in blood pressure. All these factors are closely linked to obesity and overweight and are risk factors for future chronic disease. Although the main aim of most of the studies was to measure the efficacy of mHealth interventions, they also measured other outputs that might be relevant for determining the success of the intervention, such as (1) acceptability, (2) adherence, usability, and engagement, (3) satisfaction, (4) motivation, (5) intention to continue, and (6) perceived support. All these aspects appear to affect whether an intervention will be successful. Tests and questionnaires are the most prevalent tools used for assessment, whether existing and previously validated or devised for the situation. Objective data tracking of PA performance through the mHealth-based intervention, when possible, was a common strategy for avoiding self-reported data. It appears to be highly important to gather objective data and use standardized protocols when assessing the usability and efficacy of mHealth interventions. The mHealth strategies considered to be more sophisticated usually include a higher number of elements. Although the recent strategies of peer support and gamification appear to improve efficacy by increasing engagement and motivation, there is as yet not enough evidence to state this definitively.

The acknowledgement and evaluation of comprehensive sociodemographic differences, such as race/ethnicity, socioeconomic status, and sex, are severely lacking. Future analyses of mHealth interventions should consider and, whenever possible, include eHealth literacy aspects in an effort to reduce communication inequalities across groups [80]. Unless designers and developers of health care information technologies address security challenges, benefits from health care information technology will be scarce [81]. Another aspect we have found to be lacking from mHealth evaluation studies is assessment of clinical data confidentiality.

Previously published reviews have concluded that despite a lack of evidence concerning the best use of technology in weight loss interventions, when the optimal combination of technological components is determined, technology-based interventions will be a valid tool for weight loss [82]. Others have been less optimistic and feel that future studies must use larger study samples, longer interventions, and follow-up periods [83]. One meta-systematic review concluded that despite the increasing popularity of mHealth, evidence for efficacy is still limited due to the low methodological quality of research [84]. We believe the issue may be how mHealth strategies are assessed and validated: this cannot be carried out in the same manner as research into drugs, and more adapted and/or flexible approaches are needed to explore new evaluation tools. An instrument intended to evaluate mHealth should include verification of its scientific content and mechanisms that ensure data privacy as well as safe usage. Verification of these aspects would ideally be mandatory before release and use in clinical practice. In the second phase, evaluations of effectiveness, efficacy, and usability should include user feedback, and adaptability and cost-effectiveness should also be addressed. This second phase of evaluation could be quantitative, enabling assessment of an mHealth intervention’s quality and comparison with others.

Currently, most apps used or prescribed in daily clinical practices have only received technical verification or partial clinical validation on the basis of a small group of patients.

Future research is necessary to better assess mHealth interventions in development and before clinical application. It is important to find a balance between the necessary development of mHealth, which should be characterized as disruptive, innovative, and rapid, and the imperative need to validate mHealth interventions. From a Public Health point of view, it is necessary to avoid or minimize the potential problems a new mHealth intervention might create without accurate evaluation. It has been argued that the app market regulates itself: the good persist; the bad disappear. However, in such a potentially harmful field as mHealth, there is a need for new approaches and tools, and a multidisciplinary assessment process [14,85-89].

### Limitations

One of the main limitations of this review is publication bias. References from other sources such as conferences and meetings have not been included. Although the number of scientific journals that publish mHealth-related articles has increased in recent years, there is a lot of gray literature surrounding this field that we may have missed. Moreover, only studies published in English, French, or Spanish have been included. The heterogeneity of interventions and populations and the low number of participants in many studies have made it difficult to synthesize results. Most of the studies included were deemed to be of moderate-low quality, and consequently findings need to be considered with caution. In total, 11 studies lacked a control group and therefore results cannot be attributable to the technology-based intervention alone. One must also take into account the established fact that individuals who agree to participate in intervention studies have greater motivation to change their lifestyles than the general population.

Finally, only 1 study was identified with the primary aim of assessing the safety and effectiveness of an mHealth intervention. Given awareness of safety-related issues such as a possible increase in anxiety and stress due to the use of mHealth intervention and the possible promotion of eating disorders, this is rather surprising. Furthermore, the studies reviewed largely assessed dietary habits and PA, ignoring other possible outcomes relating to body weight such as sleeping behavior. This also needs to be addressed in future research.

### Conclusions

The potential for apps to positively help users manage their obesity or overweight has yet to be attained. Studies assessing the success of mHealth interventions are remarkably heterogeneous and most have methodological limitations that leave significant room for improvement regarding quality. Further research is needed to identify all relevant criteria for assessing the efficacy of mHealth interventions in the prevention and management of overweight and obesity.

## Appendix

Multimedia Appendix 1 Search strategy.

Multimedia Appendix 2 Excluded publications.

Multimedia Appendix 3 Characteristics of the selected studies.

Multimedia Appendix 4 Elements included in the mobile health interventions of the selected studies.

Multimedia Appendix 5 Output tools and results from the selected studies.

Multimedia Appendix 6 Main outcome results from the selected studies.

## Abbreviations

BMI: body mass index
eHealth: electronic health
IPAQ: International Physical Activity Questionnaire
mHealth: mobile health
PA: physical activity
RCT: randomized control trial
SIGN: Scottish Intercollegiate Guidelines Network
WHO: World Health Organization

## References

[1] Garg SK, Maurer H, Reed K, Selagamsetty R (2014). Diabetes and cancer: two diseases with obesity as a common risk factor. Diabetes Obes Metab.

[2] Gittelsohn J, Trude A (2017). Diabetes and obesity prevention: changing the food environment in low-income settings. Nutr Rev.

[3] Khaylis A, Yiaslas T, Bergstrom J, Gore-Felton C (2010). A review of efficacious technology-based weight-loss interventions: five key components. Telemed J E Health.

[4] Aguilar-Martínez A, Solé-Sedeño JM, Mancebo-Moreno G, Medina FX, Carreras-Collado R, Saigí-Rubió F (2014). Use of mobile phones as a tool for weight loss: a systematic review. J Telemed Telecare.

[5] World Health Organization (2011). Mhealth: New Horizons for Health Through Mobile Technologies: Second Global Survey on Ehealth.

[6] McKinstry B, Hanley J, Wild S, Pagliari C, Paterson M, Lewis S, Sheikh A, Krishan A, Stoddart A, Padfield P (2013). Telemonitoring based service redesign for the management of uncontrolled hypertension: multicentre randomised controlled trial. Br Med J.

[7] (2016). Research2Guidance.

[8] Chen J, Cade JE, Allman-Farinelli M (2015). The most popular smartphone apps for weight loss: a quality assessment. JMIR Mhealth Uhealth.

[9] Ganesan AN, Louise J, Horsfall M, Bilsborough SA, Hendriks J, McGavigan AD, Selvanayagam JB, Chew DP (2016). International mobile-health intervention on physical activity, sitting, and weight: the Stepathlon cardiovascular health study. J Am Coll Cardiol.

[10] Laing BY, Mangione CM, Tseng CH, Leng M, Vaisberg E, Mahida M, Bholat M, Glazier E, Morisky DE, Bell DS (2014). Effectiveness of a smartphone application for weight loss compared with usual care in overweight primary care patients: a randomized, controlled trial. Ann Intern Med.

[11] Holmen H, Torbjørnsen A, Wahl AK, Jenum AK, Småstuen MC, Arsand E, Ribu L (2014). A mobile health intervention for self-management and lifestyle change for persons with type 2 diabetes, part 2: one-year results from the Norwegian randomized controlled trial RENEWING HEALTH. JMIR Mhealth Uhealth.

[12] Main C, Moxham T, Wyatt JC, Kay J, Anderson R, Stein K (2010). Computerised decision support systems in order communication for diagnostic, screening or monitoring test ordering: systematic reviews of the effects and cost-effectiveness of systems. Health Technol Assess.

[13] Azar KM, Lesser LI, Laing BY, Stephens J, Aurora MS, Burke LE, Palaniappan LP (2013). Mobile applications for weight management: theory-based content analysis. Am J Prev Med.

[14] Pham Q, Wiljer D, Cafazzo JA (2016). Beyond the randomized controlled trial: a review of alternatives in mhealth clinical trial methods. JMIR Mhealth Uhealth.

[15] Bradway M, Carrion C, Vallespin B, Saadatfard O, Puigdomènech E, Espallargues M, Kotzeva A (2017). mHealth assessment: conceptualization of a global framework. JMIR Mhealth Uhealth.

[16] World Health Organization.

[17] Agarwal S, LeFevre AE, Lee J, L'Engle K, Mehl G, Sinha C, Labrique A, WHO mHealth Technical Evidence Review Group (2016). Guidelines for reporting of health interventions using mobile phones: mobile health (mHealth) evidence reporting and assessment (mERA) checklist. Br Med J.

[18] (2015). Food and Drug Administration.

[19] (2014). European Commission.

[20] Carrion C, Garcia-Lorda P, Zamora A, Paluzié G, Moharra M, Puigdomènech E (2018). PROSPERO - University of York.

[21] Moher D, Liberati A, Tetzlaff J, Altman DG, PRISMA Group (2009). Preferred reporting items for systematic reviews and meta-analyses: the PRISMA statement. PLoS Med.

[22] (2015). Scottish Intercollegiate Guidelines Network (SIGN).

[23] Spook J, Paulussen T, Kok G, van Empelen P (2016). Evaluation of a serious self-regulation game intervention for overweight-related behaviors ('Balance It'): a pilot study. J Med Internet Res.

[24] Carter MC, Burley VJ, Nykjaer C, Cade JE (2013). Adherence to a smartphone application for weight loss compared to website and paper diary: pilot randomized controlled trial. J Med Internet Res.

[25] Hurkmans E, Matthys C, Bogaerts A, Scheys L, Devloo K, Seghers J (2018). Face-to-face versus mobile versus blended weight loss program: randomized clinical trial. JMIR Mhealth Uhealth.

[26] He C, Wu S, Zhao Y, Li Z, Zhang Y, Le J, Wang L, Wan S, Li C, Li Y, Sun X (2017). Social media-promoted weight loss among an occupational population: cohort study using a WeChat mobile phone app-based campaign. J Med Internet Res.

[27] Aschbrenner KA, Naslund JA, Shevenell M, Kinney E, Bartels SJ (2016). A pilot study of a peer-group lifestyle intervention enhanced with mhealth technology and social media for adults with serious mental illness. J Nerv Ment Dis.

[28] Martin SS, Feldman DI, Blumenthal RS, Jones SR, Post WS, McKibben RA, Michos ED, Ndumele CE, Ratchford EV, Coresh J, Blaha MJ (2015). mActive: a randomized clinical trial of an automated mhealth intervention for physical activity promotion. J Am Heart Assoc.

[29] Michaelides A, Raby C, Wood M, Farr K, Toro-Ramos T (2016). Weight loss efficacy of a novel mobile diabetes prevention program delivery platform with human coaching. BMJ Open Diabetes Res Care.

[30] Block G, Azar KM, Romanelli RJ, Block TJ, Hopkins D, Carpenter HA, Dolginsky MS, Hudes ML, Palaniappan LP, Block CH (2015). Diabetes prevention and weight loss with a fully automated behavioral intervention by email, web, and mobile phone: a randomized controlled trial among persons with prediabetes. J Med Internet Res.

[31] McCarroll ML, Armbruster S, Pohle-Krauza RJ, Lyzen AM, Min S, Nash DW, Roulette GD, Andrews SJ, von Gruenigen VE (2015). Feasibility of a lifestyle intervention for overweight/obese endometrial and breast cancer survivors using an interactive mobile application. Gynecol Oncol.

[32] Quintiliani LM, Mann DM, Puputti M, Quinn E, Bowen DJ (2016). Pilot and feasibility test of a mobile health-supported behavioral counseling intervention for weight management among breast cancer survivors. JMIR Cancer.

[33] Oh B, Cho B, Han MK, Choi H, Lee MN, Kang HC, Lee CH, Yun H, Kim Y (2015). The effectiveness of mobile phone-based care for weight control in metabolic syndrome patients: randomized controlled trial. JMIR Mhealth Uhealth.

[34] Svetkey LP, Batch BC, Lin P, Intille SS, Corsino L, Tyson CC, Bosworth HB, Grambow SC, Voils C, Loria C, Gallis JA, Schwager J, Bennett GG, Bennett GB (2015). Cell phone intervention for you (CITY): a randomized, controlled trial of behavioral weight loss intervention for young adults using mobile technology. Obesity (Silver Spring).

[35] Lee W, Chae YM, Kim S, Ho SH, Choi I (2010). Evaluation of a mobile phone-based diet game for weight control. J Telemed Telecare.

[36] Finkelstein J, Bedra M, Li X, Wood J, Ouyang P (2015). Mobile app to reduce inactivity in sedentary overweight women. Stud Health Technol Inform.

[37] Pretlow RA, Stock CM, Allison S, Roeger L (2015). Treatment of child/adolescent obesity using the addiction model: a smartphone app pilot study. Child Obes.

[38] Hutchesson MJ, Morgan PJ, Callister R, Pranata I, Skinner G, Collins CE (2016). Be Positive Be Healthe: development and implementation of a targeted e-health weight loss program for young women. Telemed J E Health.

[39] Partridge SR, McGeechan K, Hebden L, Balestracci K, Wong AT, Denney-Wilson E, Harris MF, Phongsavan P, Bauman A, Allman-Farinelli M (2015). Effectiveness of a mhealth lifestyle program with telephone support (TXT2BFiT) to prevent unhealthy weight gain in young adults: randomized controlled trial. JMIR Mhealth Uhealth.

[40] Lee J, Kim J (2016). Development and efficacy testing of a social network-based competitive application for weight loss. Telemed J E Health.

[41] Naimark JS, Madar Z, Shahar DR (2015). The impact of a web-based app (eBalance) in promoting healthy lifestyles: randomized controlled trial. J Med Internet Res.

[42] Mummah S, Robinson TN, Mathur M, Farzinkhou S, Sutton S, Gardner CD (2017). Effect of a mobile app intervention on vegetable consumption in overweight adults: a randomized controlled trial. Int J Behav Nutr Phys Act.

[43] Diabetes Prevention Program (DPP) Research Group (2002). The Diabetes Prevention Program (DPP): description of lifestyle intervention. Diabetes Care.

[44] Wadden TA, West DS, Delahanty L, Jakicic J, Rejeski J, Williamson D, Berkowitz RI, Kelley DE, Tomchee C, Hill JO, Kumanyika S, Look AHEAD Research Group (2006). The Look AHEAD study: a description of the lifestyle intervention and the evidence supporting it. Obesity (Silver Spring).

[45] Sauro J (2011). MeasuringU.

[46] Jung Y (2008). No verification on the participation behavior model of participants in leisure sport and exercise. Korean J Sport Psychol.

[47] Yu J (2011). The Relationship Between Fun Factor, Exercise Immersion and Participation in Women's Leisure Dance. Graduate School of Kyunghee University.

[48] Parmenter K, Wardle J (1999). Development of a general nutrition knowledge questionnaire for adults. Eur J Clin Nutr.

[49] Caron J (2013). [A validation of the social provisions scale: the SPS-10 items]. Sante Ment Que.

[50] World Health Organization (2000). Obesity: preventing and managing the global epidemic. Report of a WHO consultation. World Health Organ Tech Rep Ser.

[51] Thomas JG, Wing RR (2013). Health-e-call, a smartphone-assisted behavioral obesity treatment: pilot study. JMIR Mhealth Uhealth.

[52] Jensen CD, Duncombe KM, Lott MA, Hunsaker SL, Duraccio KM, Woolford SJ (2016). An evaluation of a smartphone-assisted behavioral weight control intervention for adolescents: pilot study. JMIR Mhealth Uhealth.

[53] Mao AY, Chen C, Magana C, Barajas KC, Olayiwola JN (2017). A mobile phone-based health coaching intervention for weight loss and blood pressure reduction in a national payer population: a retrospective study. JMIR Mhealth Uhealth.

[54] Shahar DR, Henkin Y, Rozen GS, Adler D, Levy O, Safra C, Itzhak B, Golan R, Shai I (2009). A controlled intervention study of changing health-providers' attitudes toward personal lifestyle habits and health-promotion skills. Nutrition.

[55] Attkisson CC, Greenfield TK, Maruish ME (2004). The UCSF client satisfaction scales: I. The client satisfaction questionnaire-8. The Use of Psychological Testing for Treatment Planning and Outcomes Assessment: Instruments for Adults.

[56] Lim SA, Kang SE (2013). Development and validation study of the achievement motivation scale. Korean J Educ Psychol.

[57] Bond DS, Thomas JG, Raynor HA, Moon J, Sieling J, Trautvetter J, Leblond T, Wing RR (2014). B-MOBILE--a smartphone-based intervention to reduce sedentary time in overweight/obese individuals: a within-subjects experimental trial. PLoS One.

[58] Craig CL, Marshall AL, Sjöström M, Bauman AE, Booth ML, Ainsworth BE, Pratt M, Ekelund U, Yngve A, Sallis JF, Oja P (2003). International physical activity questionnaire: 12-country reliability and validity. Med Sci Sports Exerc.

[59] Lee PH, Macfarlane DJ, Lam TH, Stewart SM (2011). Validity of the international physical activity questionnaire short form (IPAQ-SF): a systematic review. Int J Behav Nutr Phys Act.

[60] Paffenbarger RS, Hyde RT, Wing AL, Hsieh CC (1986). Physical activity, all-cause mortality, and longevity of college alumni. N Engl J Med.

[61] Larsson UE, Reynisdottir S (2008). The six-minute walk test in outpatients with obesity: reproducibility and known group validity. Physiother Res Int.

[62] Smith KJ, McNaughton SA, Gall SL, Blizzard L, Dwyer T, Venn AJ (2009). Takeaway food consumption and its associations with diet quality and abdominal obesity: a cross-sectional study of young adults. Int J Behav Nutr Phys Act.

[63] Resnicow K, Odom E, Wang T, Dudley WN, Mitchell D, Vaughan R, Jackson A, Baranowski T (2000). Validation of three food frequency questionnaires and 24-hour recalls with serum carotenoid levels in a sample of African-American adults. Am J Epidemiol.

[64] Block G, Woods M, Potosky A, Clifford C (1990). Validation of a self-administered diet history questionnaire using multiple diet records. J Clin Epidemiol.

[65] Fung TT, Chiuve SE, McCullough ML, Rexrode KM, Logroscino G, Hu FB (2008). Adherence to a DASH-style diet and risk of coronary heart disease and stroke in women. Arch Intern Med.

[66] (2001). Australian Institute of Health and Welfare.

[67] Rifas-Shiman SL, Willett WC, Lobb R, Kotch J, Dart C, Gillman MW (2001). PrimeScreen, a brief dietary screening tool: reproducibility and comparability with both a longer food frequency questionnaire and biomarkers. Public Health Nutr.

[68] Hedrick VE, Savla J, Comber DL, Flack KD, Estabrooks PA, Nsiah-Kumi PA, Ortmeier S, Davy BM (2012). Development of a brief questionnaire to assess habitual beverage intake (BEVQ-15): sugar-sweetened beverages and total beverage energy intake. J Acad Nutr Diet.

[69] Schröder H, Fitó M, Estruch R, Martínez-González MA, Corella D, Salas-Salvadó J, Lamuela-Raventós R, Ros E, Salaverría I, Fiol M, Lapetra J, Vinyoles E, Gómez-Gracia E, Lahoz C, Serra-Majem L, Pintó X, Ruiz-Gutierrez V, Covas MI (2011). A short screener is valid for assessing Mediterranean diet adherence among older Spanish men and women. J Nutr.

[70] (2003). Nurses' Health Study.

[71] Matthys C, Meulemans A, van der Schueren B (2015). Development and validation of general FFQ for use in clinical practice. Ann Nutr Metab.

[72] Cella DF, Tulsky DS, Gray G, Sarafian B, Linn E, Bonomi A, Silberman M, Yellen SB, Winicour P, Brannon J (1993). The functional assessment of cancer therapy scale: development and validation of the general measure. J Clin Oncol.

[73] Brucker PS, Yost K, Cashy J, Webster K, Cella D (2005). General population and cancer patient norms for the functional assessment of cancer therapy-general (FACT-G). Eval Health Prof.

[74] Partridge SR, Allman-Farinelli M, McGeechan K, Balestracci K, Wong AT, Hebden L, Harris MF, Bauman A, Phongsavan P (2016). Process evaluation of TXT2BFiT: a multi-component mHealth randomised controlled trial to prevent weight gain in young adults. Int J Behav Nutr Phys Act.

[75] Gomez-Marcos MA, Patino-Alonso MC, Recio-Rodriguez JI, Agudo-Conde C, Romaguera-Bosch M, Magdalena-Gonzalez O, Gomez-Arranz A, Mendizabal-Gallastegui N, Fernandez-Diez JA, Gomez-Sanchez L, Maderuelo-Fernandez JA, Rodriguez-Sanchez E, Garcia-Ortiz L, On Behalf the EVIDENT Investigators 11 (2018). Short- and long-term effectiveness of a smartphone application for improving measures of adiposity: a randomised clinical trial - EVIDENT II study. Eur J Cardiovasc Nurs.

[76] Fukuoka Y, Gay CL, Joiner KL, Vittinghoff E (2015). A novel diabetes prevention intervention using a mobile app: a randomized controlled trial with overweight adults at risk. Am J Prev Med.

[77] Willey S, Walsh JK (2016). Outcomes of a mobile health coaching platform: 12-week results of a single-arm longitudinal study. JMIR Mhealth Uhealth.

[78] Nollen NL, Mayo MS, Carlson SE, Rapoff MA, Goggin KJ, Ellerbeck EF (2014). Mobile technology for obesity prevention: a randomized pilot study in racial- and ethnic-minority girls. Am J Prev Med.

[79] Garcia-Ortiz L, Recio-Rodriguez JI, Agudo-Conde C, Patino-Alonso MC, Maderuelo-Fernandez J, Gento IR, Puig EP, Gonzalez-Viejo N, Arietaleanizbeaskoa MS, Schmolling-Guinovart Y, Gomez-Marcos MA, Rodriguez-Sanchez E, EVIDENT Investigators Group, Mobilizing Minds Research Group (2018). Long-term effectiveness of a smartphone app for improving healthy lifestyles in general population in primary care: randomized controlled trial (EVIDENT II study). JMIR Mhealth Uhealth.

[80] Kontos E, Blake KD, Chou WS, Prestin A (2014). Predictors of ehealth usage: insights on the digital divide from the health information national trends survey 2012. J Med Internet Res.

[81] Kotz D, Fu K, Gunter C, Rubin A (2015). Security for mobile and cloud frontiers in healthcare. Commun ACM.

[82] Raaijmakers LC, Pouwels S, Berghuis KA, Nienhuijs SW (2015). Technology-based interventions in the treatment of overweight and obesity: a systematic review. Appetite.

[83] Wang Y, Xue H, Huang Y, Huang L, Zhang D (2017). A systematic review of application and effectiveness of mhealth interventions for obesity and diabetes treatment and self-management. Adv Nutr.

[84] Marcolino MS, Oliveira JA, D'Agostino M, Ribeiro AL, Alkmim MB, Novillo-Ortiz D (2018). The impact of mhealth interventions: systematic review of systematic reviews. JMIR Mhealth Uhealth.

[85] Dipankui MT, Gagnon MP, Desmartis M, Légaré F, Piron F, Gagnon J, Rhiands M, Coulombe M (2015). Evaluation of patient involvement in a health technology assessment. Int J Technol Assess Health Care.

[86] Moja L, Kwag KH, Lytras T, Bertizzolo L, Brandt L, Pecoraro V, Rigon G, Vaona A, Ruggiero F, Mangia M, Iorio A, Kunnamo I, Bonovas S (2014). Effectiveness of computerized decision support systems linked to electronic health records: a systematic review and meta-analysis. Am J Public Health.

[87] Lilford RJ, Girling AJ, Sheikh A, Coleman JJ, Chilton PJ, Burn SL, Jenkinson DJ, Blake L, Hemming K (2014). Protocol for evaluation of the cost-effectiveness of ePrescribing systems and candidate prototype for other related health information technologies. BMC Health Serv Res.

[88] Cortés MA, Cuenca MR, Verdugo RM, Cidoncha EC (2014). High quantity but limited quality in healthcare applications intended for HIV-infected patients. Telemed J E Health.

[89] Nilsen W American Association for the Advancement of Science.

