# Supplementary material for: Assessment of the Efficacy, Safety, and Effectiveness of Weight Control and Obesity Management Mobile Health Interventions: Systematic Review
Source: JMIR Mhealth Uhealth. 2019 Oct 25;7(10):e12612. doi: 10.2196/12612 (PMC6913727; doi:10.2196/12612)
Supplement: Multimedia Appendix 1 [file mhealth_v7i10e12612_app1.pdf]

Search Strategy: Obesity and overweight management mhealth apps

PubMed

((app OR application)) AND ((((mHealth OR eHealth)) AND (overweight OR obesity)) AND (Efficacy OR security OR safety OR effectiveness OR evaluation))

Scopus

TITLE-ABS-KEY (((app OR application)) AND ((((mhealth OR ehealth)) AND (overweight OR obesity)) AND (efficacy OR security OR safety OR effectiveness OR evaluation)))

PsycINFO (APA)

Any Field : ((app *OR* Any Field : application)) *AND* Any Field : ((((mHealth *OR* Any Field : eHealth)) *AND* Any Field : (overweight *OR* Any Field : obesity)) *AND* Any Field : (Efficacy *OR* Any Field : security *OR* Any Field : safety *OR* Any Field : effectiveness *OR* Any Field : evaluation))

ClinicalTrials.gov

((app OR application)) AND ((((mHealth OR eHealth)) AND (overweight OR obesity)) AND (Efficacy OR security OR safety OR effectiveness OR evaluation))

Cochrane Central Register of Controlled Trials (CENTRAL)

(app or application) and (((mHealth or eHealth) and (overweight or obesity)) and (Efficacy or security or safety or effectiveness or evaluation))

Clinicaltrialsregister.eu

overweight AND health

UK tripdatabase

(app or application) and (((mHealth or eHealth) and (overweight or obesity)) and (Efficacy or security or safety or effectiveness or evaluation))
