# Supplementary material for: Assessment of the Efficacy, Safety, and Effectiveness of Weight Control and Obesity Management Mobile Health Interventions: Systematic Review
Source: JMIR Mhealth Uhealth. 2019 Oct 25;7(10):e12612. doi: 10.2196/12612 (PMC6913727; doi:10.2196/12612)
Supplement: Multimedia Appendix 2 [file mhealth_v7i10e12612_app2.pdf]

MULTIMEDIA APPENDIX 2

Excluded studies after full-text review and reasons why they were excluded:

Study	Reason for exclusion
Bardus M, van Beurden SB, Smith JR, Abraham C. A review and content analysis of engagement, functionality, aesthetics, information quality, and change techniques in the most popular commercial apps for weight management. Int Journal of Behavioral Nutrition and Physical Activity 2016; PMID: 26964880	Outcomes: Engagement, functionality, aesthetics and information quality.<br><br>*Final outcomes other than efficacy, safety or effectiveness.*
González C, Herrero P, Cubero JM, Iniesta JM, Hernando ME, García-Sáez G, Serrano AJ, Martinez-Sarriegui I, Perez-Gandia C, Gómez EJ, Rubinat E, Alcantara V, Brugués E, Chico A, Mato E, Bell O, Corcoy R, de Leiva A. PREDIRCAM eHealth platform for individualized telemedical assistance for lifestyle modification in the treatment of obesity, diabetes, and cardiometabolic risk prevention: a pilot study (PREDIRCAM 1). Journal of Diabetes Science and Technology 2013; PMID: 23911170	Outcome: Feasibility of the platform.<br><br>*Final outcomes other than efficacy, safety or effectiveness.*
Naslund JA, Aschbrenner KA, Barre LK, Bartels SJ. Feasibility of popular m-health technologies for activity tracking among individuals with serious mental illness. Telemed J E Health 2015. PMID: 25536190	Outcome: Feasibility<br><br>*Final outcomes other than efficacy, safety or effectiveness.*
Chen J, Cade JE, Allman-Farinelli M. The Most Popular Smartphone Apps for Weight Loss: A Quality Assessment. JMIR Mhealth Uhealth 2015. PMID: 26678569	Outcome: quality assessment<br><br>*Final outcomes other than efficacy, safety or effectiveness.*
Oliver E, Baños RM, Cebolla A, Lurbe E, Alvarez-Pitti J, Botella C. An electronic system (PDA) to record dietary and physical activity in obese adolescents; data about efficiency and feasibility. Nutr Hosp. 2013. PMID: 24506361	Outcome: Feasibility<br><br>*Final outcomes other than efficacy, safety or effectiveness.*
Walsh JC, Corbett T, Hogan M, Duggan J, McNamara A. An mHealth Intervention Using a Smartphone App to Increase Walking Behavior in Young Adults: A Pilot Study. JMIR Mhealth Uhealth 2016. PMID: 27658677	Outcome: efficacy of Accupedo-Pro Pedometer app in increasing number of daily steps.<br><br>*Final outcomes other than efficacy, safety or effectiveness.*
Mundi MS, Lorentz PA, Grothe K, Kellogg TA, Collazo-Clavell ML. Feasibility of Smartphone-Based Education Modules and Ecological Momentary Assessment/Intervention in Pre-bariatric Surgery Patients. Obesity Surgery 2015. PMID: 25702141	Outcome: Feasibility of using a smartphone app in the education.<br><br>*Final outcomes other than efficacy, safety or effectiveness.*
Martin CK, Gilmore LA, Apolzan JW, Myers CA, Thomas DM, Redman LM. SmartLoss: A Personalized Mobile Health Intervention for Weight Management and Health Promotion. JMIR mHealth and uHealth 2016. PMID: 26983937	Outcome: To describe SmartLoss, a semi automated mHealth platform for weight loss<br><br>*Final outcomes other than efficacy, safety or effectiveness.*
Naslund, JA, Aschbrenner KA, Bartels S. Wearable Devices and Smartphones for Activity Tracking Among People with Serious Mental Illness. Mental Health and Physical Activity 2016. PMID: 27134654	Outcome: acceptability and usability<br><br>*Final outcomes other than efficacy, safety or effectiveness*
Skau JK, Nordin AB, Cheah JC, Ali R, Zainal R, Aris T, Ali ZM, Matzen	*Published study protocol*

P, Biesma R, Aagaard-Hansen J, Hanson MA, Norris S. A complex behavioural change intervention to reduce the risk of diabetes and prediabetes in the pre-conception period in Malaysia: study protocol for a randomised controlled trial. Trials 2016. PMID: 27117703	
Patrick K, Marshall SJ, Davila EP, Kolodziejczyk JK. Fowler J, Calfas KJ, Huang J, Rock CL, Griswold W, Gupta A, Merchant G, Norman GJ, Raab F, Donohue M, Fogg BJ, Robinson TN. Design and Implementation of a Randomized Controlled Socialand Mobile Weight Loss Trial for Young Adults (project SMART). Contemp Clin Trials 2014. PMID: 24215774	*Published study protocol*
Pellegrini CA, Hoffman SA, Collins LM, Spring B. Optimization of remotely delivered intensive lifestyle treatment for obesity using the Multiphase Optimization Strategy. Contemp Clin Trials 2014. PMID: 24846621	*Published study protocol*
Granado-Font E, Flores-Mateo G, Sorlí-Aguilar M, Montaña-Carreras X, Ferre-Grau C, Barrera-Uriarte M, Oriol-Colominas E, Rey-Reñones C, Caules I, Satué-Gracia E. Effectiveness of a Smartphone application and wearable device for weight loss in overweight or obese primary care patients: protocol for a randomised controlled trial. BMC Public Health 2015. PMID: 26041131	*Published study protocol*
Boh B, Lemmens L, Jansen A, Nederkoorn C, Kerkhofs V, Spanakis G, Weiss G, Roefs A. An Ecological Momentary Intervention for weight loss and healthy eating via smartphone and Internet. Trials 2016. PMID: 27000058	*Published study protocol*
Shin D, Joh H, Yun J, Kwon H, Lee H, Min H, Shin J, Chung W, Park J, Cho B. Design and baseline characteristics of participants in the Enhancing Physical Activity and Reducing Obesity through Smartcare and Financial Incentives (EPAROSFI): A pilot randomized controlled trial. Contemporary Clinical Trials 2016. PMID: 26744232	*Published study protocol*
Ifejika NL, Noser EA, Grotta JC, Savitz SI. Swipe out Stroke: Feasibility and efficacy of using a smart-phone based mobile application to improve compliance with weight loss in obese minority stroke patients and their carers. International Journal of Stroke 2016; PMID: 26956032	*Published study protocol*
Delisle C, Sandin S, Forsum E, Henriksson H, Trolle-Lagerros Y, Larsson C, Maddison R, Ortega FB, Ruiz JR, Silfvernagel K, Timpka T, Löf M. A web- and mobile phone-based intervention to prevent obesity in 4-year-olds (MINISTOP): a population-based randomized controlled trial. BMC Public Health 2015; PMID: 25886009	*Published study protocol*
Chen JL, Kwan M, Mac A, NC Chin, Liu K. iStart Smart: A Primary-Care Based and Community Partnered Childhood Obesity Management Program for Chinese-American Children: Feasibility Study. Journal of Immigrant and Minority Health 2013. PMID: 23595264	*Out of scope: Not an mHealth intervention*
Lubans DR, Smith JJ, Plotnikoff RC, Dally KA, Okely AD, Salmon J, Morgan PJ. Assessing the sustained impact of a school-based obesity prevention program for adolescent boys: the ATLAS cluster randomized controlled trial. Int J Behav Nutr Phys Act. 2016. PMID: 27542825	*Out of scope: Not an mHealth intervention*
Shaw RJ, Bosworth HB, Silva SS, Lipkus IM, Davis LL, Sha RS,	*Out of scope: SMS intervention*

Johnson CM. A mobile health intervention to sustain weight loss. Am J Med 2013. PMID: 24050486	
Carnero-Gregorio M, Blanco-Ramos M, Obeso-Carillo GA, García-Fontán E, Álvarez-González MÁ, Cañizares-Carretero MÁ. Design and development of a nutritional assessment application for smartphones and tablets with Android OS. Nutrición Hospitalaria 2015. PMID: 25726229	*Out of scope*: nutritional calculation software.
