# Supplementary material for: Assessment of the Efficacy, Safety, and Effectiveness of Weight Control and Obesity Management Mobile Health Interventions: Systematic Review
Source: JMIR Mhealth Uhealth. 2019 Oct 25;7(10):e12612. doi: 10.2196/12612 (PMC6913727; doi:10.2196/12612)
Supplement: Multimedia Appendix 3 [file mhealth_v7i10e12612_app3.pdf]

Multimedia Appendix 3: Characteristics of the selected studies

Reference	Design (N)	Country	Population	Intervention	Quality	Limitations
Lee 2010 [23]	CT[a] (2 groups, 36)	The United States and Latin America	Sex: No Inf[b] Age: 28.2 (i)[c] 29.5 (c)[d] BMI[e]: 22.2 (i) 22.3 (c)	Efficacy of the SmartDiet app (6 weeks)	Moderate	No specifications about control conditions or randomization method.
Carter 2013 [24]	RCT[f] (3 groups, 128)	The United Kingdom	Sex: 77% women Age: 42 (SD 9) BMI: 34 (SD 5)	Efficacy in weight managemen t intervention by My Meal Mate app (6 months)	Low	Selection bias: motivated volunteers, different educational level according to randomization.
Thomas 2013 [25]	Single-arm pre-post (1 group, 20)	The United States	Sex: 95% women Age: 53 (SD 1.9) BMI: 36.3 (SD 1.2)	Efficacy of Daily Burn + Health-E-call app (24 weeks)	Low	Small sample size. Short-term effects (24 weeks). No control group. Potential motivational bias.
Bond 2014 [26]	Single-arm pre-post (1 group, 30)	The United States	Sex: 83% women Age: 47.5 (SD 13.5) BMI: 36.2 (SD 7.5)	Efficacy of B-MOBILE app to reduce time spent in sedentary	Low	Small sample size. Selection bias: volunteers. Possible misclassification of sedentary

				behavior (4 weeks)		behavior by the armband sensor.
Nollen 2014 [27]	RCT (2 groups, 51)	The United States	Sex: women Age: 11.3 (SD 1.6) BMI: 23.7 (SD 5.7) Low-income, ethnic minority	Efficacy of MyPal A626 behavioral change in weight control (12 weeks)	Moderate	Small sample size. Too short to appreciate behavioral change.
Block 2015 [28]	RCT (2 groups, 339)	The United States	Sex: 31.27% woman. Age: 55 (SD 8.9) BMI: 31.2 (SD 4.4) Prediabetic patients	Efficacy of Alive-PD that provides tailored behavioral support (6 months)	High	Potential selection bias: study participants were relatively well educated and mainly non-Hispanic white people.
Finkelstein 2015 [29]	RCT (2 groups, 30)	The United States	Sex: all women Age: 52 (SD 12) BMI: 37 (SD 6)	Efficacy of mHealth intervention to increase PA (8 weeks)	Moderate	No information about recruitment. Randomized crossover design study.
Fukuoka 2015 [30]	RCT (2 groups, 61)	The United States	Sex: 77% women Age: 55.2 (SD 9.0) BMI: 33.3 (SD 6.0)	Diabetes Prevention Program (monitoring of weight, caloric intake, and	Moderate	Less number of participants. Selection bias: participants willing to use a mobile app and a pedometer.

			48% were racial/ethnic minorities. Prediabetic condition.	PA[g]), with reduced number of face-to-face + home-based exercise program (5 months)		
Martin 2015 [31]	RCT (2 groups, 48)	The United States	Sex: 46% women Age: 58 (SD 8) BMI: 31 (SD 6) Patients with heart disease	Efficacy of a mActive intervention in PA performance (5 weeks)	Moderate	Limited size and scope. Different stages of the trial with different approaches when blinding participants.
McCarroll 2015 [32]	Single-arm pre-post (1 group, 50)	The United States	Sex: women Age: 58.4 (SD 10.3) BMI: 34.9 (SD 8.7) Overweight cancer survivors	Efficacy of nutrition and exercise counseling through Loose It app (1 month)	Low	Small sample size. Noncontrolled single-arm trial. Low completion rate (70%).
Oh 2015 [33]	RCT (2 groups, 422)	Korea	Sex: 46.7% (i) and 51.4% (c) women. Age: 46.78 (i) 50.35 (c)	Efficacy of SmartCare services in obese patients with	High	Potential selection bias: higher educated participants were assigned to the intervention

			BMI: 29.42 (SD 3.53) (i)<br>29.40 (SD 3.39) (c)	metabolic syndrome. Safety was also assessed (24 weeks)		group despite random selection.
Partridge 2015 [34], 2016 [35]	RCT<br>(2 groups, 214)	Australia	Sex: 61% women<br>Age: 27.7 (SD 4.9)<br>BMI: 27.1 (SD 2.5)	Efficacy and engagement with the mHealth program components of TXT2BFiT intervention (3 months + 6 follow-up months)	High	Results might have been biased since the sample was mostly well educated and from high socioeconomic areas.
Pretlow 2015 [36]	Single-arm pre-post<br>(1 group, 43)	Australia	Sex: 65% women<br>Age: 16 (SD 0.43)<br>BMI: 0.98 (SD 0.0) mean percentile.	Efficacy of mHealth intervention for weight loss based on an addiction treatment approach<br>(20 weeks)	Low	Small sample size.<br>Noncontrolled study.<br>Selection bias: participants were selected if motivated; economic compensation proportional to completion.<br>Low completion rate (63%).

Naimark 2015 [37]	RCT (2 groups, 85)	Israel	Sex: 54% women Age: 47.9 (SD 12.3) BMI: 26.2 (SD 3.9)	Efficacy of Web-based eBalance app for healthy people (14 weeks)	High	Good retention rate in both control and intervention group. Short length of the intervention.
Spook 2015 [38]	Cluster randomization trial (2 groups, 501)	The Netherlands	Sex: 62.8% woman. Age: 17.28 (SD 1.3) BMI: 21.1 (SD 3.3) Secondary school students	Efficacy of Balance It, serious self-regulation game intervention (4 weeks)	Low	Very high dropout rates (72.4%). Cluster randomized trial at school level. Short-term intervention. Self-reported outcomes.
Svetkey 2015 [39]	RCT (3 groups, 365)	The United States	Sex: 69.6% women Age (mean): 29.4 BMI (mean): 35.2	Efficacy of app or professional coach intervention to lose weight (24 months)	High	No important limitations.
Aschbrenner 2016 [40]	Single-arm pre-post (1 group, 32)	The United States	Sex: 56% women Age: 48.8 (SD 11.9) BMI: 37.7 (SD 7.9) Patients with serious	Efficacy of lifestyle change intervention (apps + wearable + Facebook) to reinforce	Low	Noncontrolled single-arm study. Small sample size.

			mental illness.	PA and healthy eating (24 weeks)		
Hutchesson 2016 [41]	Single-arm pre-post (1 group, 18)	Australia	Sex: all women<br>Age: 22.8 (SD 3.2)<br>BMI: 27.3 (SD 1.6)	Be positive—Be health program for weight loss (3 months)	Low	Single-arm study, noncontrolled.<br>Nonprobabilistic selection of participants.<br>Small sample.
Jensen 2016 [42]	Single-arm pre-post (1 group, 16)	The United States	Sex: 75% women<br>Age: 14.29 (SD 1.1)<br>BMI: 1.85 (SD 0.1)	Efficacy of mobile phone - assisted adolescent behavioral weight control intervention with Daily Burn app (24 weeks)	Low	Small sample.<br>Heterogeneity of participants.<br>No control group.
Lee 2016 [43]	Single-arm pre-post (1 group, 20)	Korea	Sex: 33% women<br>Age: 20-40<br>BMI:<br>23-25: 38.5%<br>25-30: 33.0%<br>>30: 28.5%	Develop and test efficacy of With U app and social network offline and web-based (4 weeks)	Low	Single-arm study, noncontrolled.<br>No information about selection of participants.<br>Small sample.

Michaelides 2016 [44]	Single-arm pre-post (1 group, 43)	The United States	Sex: 86% women Age: 51.5 (SD 8.3) BMI: 35.5 (SD 6) Hyperglycemic (HbA_{1c}[h] between 5.7% and 6.4%)	Efficacy of a novel mobile Diabetes Prevention Program delivery platform with human coaching (24 weeks)	Low	No control group. Small sample.
Quintiliani 2016 [45]	Single-arm pre-post (1 group, 10)	The United States	Sex: all women Age: 59 (SD 6) Overweight breast cancer survivors 50% ethnic minority group	Mobile health-supported behavioral counseling intervention for weight control (10 weeks)	Low	Single-arm study, noncontrolled. Small sample. Ownership of a smartphone as well as home Wi-Fi was required.
Willey 2016 [46]	Single-arm pre-post (1 group, 10)	The United States	Sex: All women Age: 43.5 (35-49) BMI: 31.6 (range 27.2-36.4).	Efficacy of YouPlus Health coaching platform (12 weeks)	Low	Single-arm study, noncontrolled. Small sample.
Gomez-Marcos 2017 [47] and	RCT (2 groups, 833, 415	Spain	Sex: 60.0% (i) 64.1% (c) women	mHealth intervention to improve the	High	No blinding due to the nature of the intervention.

Garcia-Ortiz 2018 [48]	(i) and 418 (c)		Age: 51.4 (SD 12.1) (i) and 52.3 (SD 12.0) (c)<br>BMI: 28.1 (SD 5.1) (i) and 27.6 (SD 4.6) (c)	adherence to a Mediterranean diet and increase of PA (3 months)		Dropout rate above 10%.<br>No guarantee other apps on PA or diet were not used.
He 2017 [49]	CT<br>(2 groups, 15,310)	China	Sex: 40.5% (i) and 66.5% (c) woman.<br>Age: 35.1 (SD 8.5) (i) and 39.0 (SD 9.5) (c)<br>BMI: No Inf.	Effectiveness of We Chat intervention (6 months)	Low	No randomization.<br>Self-reported outcomes.<br>High dropout rates in the intervention group.
Mummah 2017 [50]	RCT<br>(2 groups, 135)	The United States	Sex: 62.2% women<br>Age: 39.4 (SD 6.7) (i) 40.3 (SD 5.8) (c)<br>BMI: 28-40	Efficacy of Vegethon app to increase vegetable consumption (5 weeks)	High	No relevant limitations.
Mao 2017 [51]	CT<br>(2 groups, 1012, 763 (i) and 249 (c)	The United States	Sex: 66.7% women<br>Age: 44.6 (SD 11.3)<br>BMI: 33.5 (SD 0.2)	Efficacy of health coaching service (Vida Health app) + wireless	Low	No randomization.<br>Retrospective data.<br>Self-reported data.

				scale, pedometer, and blood pressure managemen t (5 months)		Lack of a true control group.
Hurkmans 2018 [52]	RCT (4 groups, 102)	Belgium	Sex: 84% (i) and 75% (c) women<br>Age: 44 (SD 12.4) (i) and 45 (SD 10.2) (c)<br>BMI: 32 (SD 2.0)	Efficacy in weight loss and other outcomes of an app intervention, alone or combined with face-to-face coaching (3 months)	Moderate	Small sample. Possible bias in data collection. Self-reported outcomes.

[a]CT: nonrandomized controlled trial.

[b]No Inf: no information available.

[c](i): intervention group.

[d](c): control group.

[e]BMI: body mass index (always measured as kg/m^2).

[f]RCT: randomized control trial.

[g]PA: physical activity.

[h]HbA_{1c}: hemoglobin A_{1c}.
