# Supplementary material for: Assessment of the Efficacy, Safety, and Effectiveness of Weight Control and Obesity Management Mobile Health Interventions: Systematic Review
Source: JMIR Mhealth Uhealth. 2019 Oct 25;7(10):e12612. doi: 10.2196/12612 (PMC6913727; doi:10.2196/12612)
Supplement: Multimedia Appendix 4 [file mhealth_v7i10e12612_app4.pdf]

Multimedia Appendix 4: Elements included in the mobile health interventions of the selected studies.

Mobile health intervention	Components	Elements included						
		Education	Self-monitoring	Setting goals	Feedback	Gamification	Peers network	Entertainment
SmartDiet [23]	MyPage app<br>Diet Game app	—[a]	+	+	—	+	—	—
Health-E-call [25]	Daily Burn app + Health-E-call app	+	+	+	+	—	—	—
My Meal Mate [24]	Stand-alone app	—	+	+	+	—	—	—
B-mobile [26]	Aka app + armband sensor	—	—	+	+	—	—	—
Stand-alone app [27]	Stand-alone app	—	+	+	+	—	—	—
Multicomponent [36]	App<br>Face-to-face sessions<br>Phone meetings	—	+	+	+	—	+	+
Loose It [32]	Stand-alone app	—	+	+	+	—	—	—
Multicomponent [29]	Fitbit<br>App<br>Website	—	+	+	+	—	—	—
Diabetes Prevention Program	App<br>Pedometer	+	+	—	+	—	—	+

(mDPP) [30]	Face-to-face sessions							
mActive [31]	Fitbug app Armband sensor	—	—	+	+	—	—	—
TXT2BFiT [34,35]	eVIP, ePass, eSIYP apps My Message Media Website	+	+	+	+	—	+	—
eBalance [37]	Stand-alone app	—	+	—	+	—	—	—
Cell phone intervention for you (CITY) [39]	Stand-alone app Personal coach	—	—	+	—	—	+	—
SmartCare [33]	App Body composition monitors Pedometers	+	+	+	+	—	—	—
Alive-PD [28]	App Web Email Interactive voice response Phone calls	+	+	+	+	+	+	+
Balance It [38]	Stand-alone app	+	—	+	+	+	—	+
Multicomponent [40]	Fitbit App Facebook	—	+	+	+	—	+	—

Be Positive Be Healthe [41]	Website App	—	+	+	+	—	+	+
Daily Burn [42]	Stand-alone app	+	+	+	+	—	—	—
With U [43]	App Facebook	—	—	+	—	—	+	—
Noom [44]	App Personal Health Coach	+	+	—	+	+	—	—
Multicomponent [45]	Fitbit Aria Scale Fitbit Flex Tele and face-to-face counseling	+	—	—	+	—	—	—
YouPlus Health [46]	Stand-alone app	+	+	—	+	—	—	—
Evident II [47,48]	App Pedometer	—	+	—	+	—	—	—
Vegethon [50]	Stand-alone app	—	—	+	+	—	+	—
Vida Health [51]	App Scale Pedometer Blood pressure cuff	+	—	—	+	—	—	—
We Chat [49]	Stand-alone app	+	—	—	+	+	+	—
B-slim [52]	Stand-alone app	+	+	+	—	—	+	—

[a]—: data not applicable
