# Supplementary material for: Assessment of the Efficacy, Safety, and Effectiveness of Weight Control and Obesity Management Mobile Health Interventions: Systematic Review
Source: JMIR Mhealth Uhealth. 2019 Oct 25;7(10):e12612. doi: 10.2196/12612 (PMC6913727; doi:10.2196/12612)
Supplement: Multimedia Appendix 5 [file mhealth_v7i10e12612_app5.pdf]

Multimedia Appendix 5: Output tools and results from the selected studies.

Output	Reference	Tool	Result
Acceptability	Carter 2013 [24]	Evaluation survey	At 6 months, 63.2% of smartphone participants were satisfied or very satisfied compared with 50.0% in the diary group and 42.1% in the website group (*P*=.05).
	Finkelstein 2015 [29]	Focus group	A majority of the participants expressed high acceptance of the mobile app and indicated willingness to use it in the future.
	Hutchesson 2016 [41]	Evaluation survey<br>Objective data tracking participants' performance	• Mean satisfaction was 3.4 (maximum of 5).<br>• There were 22 posts to the discussion forum.<br>• One-third of participants (n=6) added at least 1 post to the discussion forum.
	Quintiliani 2016 [45]	Open questions survey	• Nine out of 10 participants responded that it is *very likely* that they would participate again or recommend the program to others.<br>• However, 7 out of 10 participants responded that it is *somewhat unlikely* or *not at all likely* that they would participate again if they had to pay for the program.
Usability/adherence/engagement	Lee 2010 [23]	Data tracking	The mean number of transmissions was 12.4 per patient.
	Carter 2013 [24]	Intervention use	Intervention usage was highest in the smartphone group: mean of 92 (SD 67) days completed compared with 29 (SD 39) days in the diary group and 35 (SD 44) days in the website group.
	Thomas 2013 [25]	Data tracking	Participants adhered to self-monitoring at 90.8 (3.3%) at 12 weeks and 84.9 (4.0%) at 24 weeks. Participants were considered to be adhering if recording daily body weight and at least 3 meals or food intake per day.

	Nollen 2014 [27]	Time and date tracking	Girls used the program on 63% of days, responded to 42% of prompts, and earned an average of 23.9 songs.
	McCarroll 2015 [32]	Data tracking	Patients who failed to log more than 3 days in a row: 30% (15 participants).
	Spook 2015 [38]	Data tracking	Only 27.6% of the participants used the intervention.
	Partridge 2016 [35]	Semistructured telephone interviews<br>Online surveys<br>Data tracking	• Smartphone apps, resources, and community blog used by less than 25% of participants.<br>• Coaching calls, text messages, and emails were described as helpful to achieving goals.
	Safran 2015 [37]	Data tracking<br>Google analytics	• The mean frequency of use was 2.7 (SD 1.9) days a week (95% CI 2.2-3.2). The average period of use was 7.8 (SD 4.3) weeks.<br>• Self-monitoring declined over the study period. At the end of 14 weeks, 27% of users were still active on the app. The average duration of visits was 7.5 (SD 0.9) min and the average number of visits per page was 6.2 (SD 0.6).
	Svetkey 2015 [39]	Data tracking	Participants interacted with the study app an average of 4.6 times/day in the first 6 months and 0.7 times/day in the final year.
	Block 2015 [28]	Data tracking	Interaction was a median of 17 of the 24 weeks (interquartile range 14). In all, 87.1% of the participants interacted with the program in 4 or more of the 24 weeks.
	Aschbrenner 2016 [40]	Usage of Fitbit and private Facebook group	All (100%) of the participants used the Fitbit and 76% used the private Facebook group.
	Jensen 2016 [42]	Self-monitoring	• On average, participants monitored at least 2 meals on 48.3% of days during the in-person intervention (12 weeks).

			• Participants monitored at least 2 meals on 16.6% of the available days during the electronic-only intervention period (12 weeks).
	Lee 2016 [43]	System Usability Scale [55]	63 out of 100 points indicated slightly low usability (threshold is 68).
	Michaelides 2016 [44]	Data tracking	• Meals per week logged 15.3 (SD 5.1).<br>• Minutes per week of exercise 141.6 (SD 112.9).<br>• Number of group comments per week 2.1 (SD 1.8).
	Quintiliani 2016 [45]	Data tracking	Mean number of responses was 60 (SD 13), for responding to text messages; 64 (SD 7) for recording a step measurement; 45 (SD 24) for recording a weight measurement; and 43 (SD 19) for recording a sleep measurement, out of a possible 70.
	Willey 2016 [46]	Data tracking	• 100% completed tutorials.<br>• Number of questions asked 16-276 in discussion.<br>• Number of questions answered 100-276 in forums.<br>• Mean weekly opens: 5.1-18.4.
	Garcia-Ortiz 2018 [48]	Number of recorded days on the device	• 100% completed tutorials.<br>• The median use of the app was 67 days.<br>• 56.8% participants in the intervention group had high app adherence (more than 60 days).<br>• Participants with low adherence were younger (49.5 vs 52.9 years), and there was a higher proportion of smokers.
Satisfaction	Thomas 2013 [25]	Likert scale	All participants endorsed the maximum rating for satisfaction.
	Oh 2015 [33]	Likert scale	On a 1-5 scale, satisfaction was 3.92 (SD 0.85).
	Pretlow 2015 [36]	Likert scale	On a 1-5 scale, satisfaction was 3.11 (SD 0.15).

	Safran 2015 [37]	Ad hoc questionnaire based on Shahar et al [56]	• Moderate to very high recommendation for the app 93%.<br>• Satisfaction on a scale of 1-10 was 7.3 (SD 1.9).
	Jensen 2016 [42]	Client satisfaction standardized questionnaire [57] Semistructured interviews.	• Score: 20.3 out of 22.<br>• Most described the intervention favorably (86%), reporting that the intervention *worked well* or was *very helpful*.<br>• Participants enjoyed learning about nutrition and exercise (33%) and being able to meet with an expert to have their questions answered (20%).<br>• Half of the participants found the Daily Burn app to be *tedious* and *difficult to use* (53%).
	Lee 2016 [43]	Lim and Kang scale [58]	Before/after 48.7/54.2 points (*P*<.01).
	Mao 2017 [51]	Rating of the App (out of 10)	Only 43.6% of participants in the intervention group rated their satisfaction, score=9.8 (SD 0.7).
Motivation to weight loss	Bond 2014 [26]	Likert scale	90% of participants endorsed either a 4 (n=11) or 5 (n=17) indicating that the app intervention significantly increased their motivation.
	Lee 2016 [43]	Jung [59] and Yu [60] scales	A score of 15.4 (SD 1.4) out of a possible 20.
Intention to continue	Safran 2015 [37]	Self-report questionnaire based on Parmenter and Wardle [61]	Control group: no significant change (*P*=.16) but in the app group, from 76 (SD 7.5) to 79 (SD 8.7) at the end of the study (*P*=.04).
Perceived support	Aschbrenner 2016 [40]	Social Provisions Scale [62]	Weight loss significantly associated with perceived peer-group support (r=0.59, *P*=.02).

	Quintiliani 2016 [45]	Ad hoc Perceived Stress Scale	Reductions in fatigue, loss of control in eating, and perceived stress of −1.8 (SD 0.8), −0.5 (SD 0.7), and −0.4 (SD 3.3), respectively.
