# Supplementary material for: Assessment of the Efficacy, Safety, and Effectiveness of Weight Control and Obesity Management Mobile Health Interventions: Systematic Review
Source: JMIR Mhealth Uhealth. 2019 Oct 25;7(10):e12612. doi: 10.2196/12612 (PMC6913727; doi:10.2196/12612)
Supplement: Multimedia Appendix 6 [file mhealth_v7i10e12612_app6.pdf]

Multimedia Appendix 6: Main outcome results from the selected studies.

Outcome	Reference	Tool	Result
Weight reduction /BMI[a]	Lee 2010 [23]	Bioelectrical impedance analysis with InBody 720	Reduction in weight in the intervention group 1.9 (ss)[b] and 0.5 kg in control group (ns).[c]
	Carter 2013 [24]	Weight by Watchers 8958U Body Analyser Scale portable.<br>Height portable stadiometer to the nearest 0.1 cm	• Weight reduction: ITT[d] mean in app group 4.6 kg (ss); in the diary group 2.9 kg (ss); and in the website group 1.3 kg (ns).<br>• BMI[d] ITT mean change at 6 months −1.6 kg/m^2 (ss) in the app group; −1.0 kg/m^2 (ss) in the diary group; and −0.5 kg/m^2 (ns). Difference (*P*=.004).<br>• Follow-up weight between the groups at 6 months (ss).
	Thomas 2013 [25]	Digital scale and stadiometer.	Weight reduction: 10.9 (1.1) kg.[e]
	Block 2015 [28]	Nonspecified	• Weight loss: intervention group 3.26 (95% CI −3.26 to −3.25) kg; control group 1.26 (95% CI −1.27 to −1.26) kg (*P*<.001).<br>• BMI loss: intervention group 1.05 (95% CI −1.06 to −1.05); control group 0.9 (95% CI −0.39 to −0.38) (*P*<.001).
	Fukuoka 2015 [30]	Tanita WB-110 digital electronic scale and conventional stadiometer	• Intervention group lost an average of 6.2 kg between baseline and 5-month follow-up compared with

			control group which gained 0.3 kg (P<.001).<br>• Mean BMI decreased in the intervention group with almost no change among controls (P<.001).
	McCarroll 2015 [32]	499KI. Health O Meter Professional Digital Column Scale	BMI decreased from 34.9 (8.7) kg/m^2 to 33.9 (8.4) kg/m^2 (P<.001).[e]
	Oh 2015 [33]	Bioelectrical impedance analysis with InBody U20	• Weight reduction was 2.21 (3.60) kg in the intervention group and 0.77 (2.77) kg in the control group (P<.001).[e]<br>• BMI reduction was 0.86 (1.32) kg/m^2 and 0.33 (1.05) kg/m^2 (P<.001), respectively.[e]
	Pretlow 2015 [36]	Health-O-meter stadiometer (Continental Scale Corp.) and self-calibrating 500-pound capacity Faribanks digital scale.	• Males 13.3% over BMI.<br>• Females 3.8% over BMI.
	Safran 2015 [37]	Portable digital scale (Beurer GmbH & Co. KG).	• The app users lost more weight compared to the control group: −1.4 (0.4) kg versus −0.13 (0.4) kg (P=.03).[e]<br>• The mean BMI change was −0.5 (0.1) kg/m^2 in the app group, but only −0.03 (0.1) kg/m^2 in the control group (P=.03).[e]
	Svetkey 2015 [39]	Weight: in high-quality calibrated digital scale.	• All groups lost weight at 6, 12, and 24 months. No significant differences between the 3 groups at 24 months.<br>• Personal coach greatest mean weight loss at 6 (−3.1 kg) and 12 months

			(−2.1 kg) than app (6 months=−2.2 kg, 12 months=−2.1 kg) (*P*<.05).
	Aschbrenner 2016 [40]	Weight loss calculated as the proportion that achieved clinically significant reduction of ≥5% from baseline weight.	• Weight loss: 72% participants lost weight, 28% achieving clinically significant weight loss.<br>• Weight loss of 7.8 (12) kg.[e]<br>• BMI decrease of 1.3 (2.0) kg/m^2.[e]
	Hutchesson 2016 [41]	Bioelectrical impedance analysis with InBody 720	Reduction in weight in 1.5 (2.4) kg; (*P*=.02).[e]
	Jensen 2016 [42]	Weight with a digital scale (Seca 869) and height using a portable stadiometer (Seca 217)	• Significant reduction in weight during face-to-face + app intervention (*P*=.04).<br>• Back to initial weight after only online intervention.
	Lee 2016 [43]	Bioelectrical impedance analysis with InBody 720	Weight measures: Before: 80.2 kg, After 79.3 kg (*P*<.001).
	Michaelides 2016 [44]	No information	• Weight loss at 16 and 24 weeks was significant.<br>• A rate of 64% of completers losing over 5% body weight.
	Partridge 2016 [35]	Self-reported data<br>Standardized protocol for weight and height [63]	• No difference between self-reported or measured data.<br>• Weight loss: 2.2 kg (0.8-3.6), *P*=.005.<br>• BMI loss: 0.5 13 kg/m^2 (0.1-1.0), *P*=.02.
	Quintiliani 2016 [45]	Scale not specified	Mean weight reduction in 1.5 (3.5) kg.[e]
	Willey 2016 [46]	Single calibrated scale at physician's office	Reduction of 6.1 kg representing 7.3% of baseline, *P*=.005.

	Gomez-Marcos 2017 [47]	Seca 770 scale, Seca 222 height rod.	At 3 months, there were no significant differences in baseline measurements in the overall population.
	He 2017 [49]	Auto-reported weight	No significant decrease of weights between groups: control group 1.78 (2.96) kg and intervention group 2.09 (3.43) kg.[e]
	Mao 2017 [51]	Weight via a Bluetooth scale. Some participants self-enter data	Mean weight loss at 4 months in intervention group: −3.2 (0.2) kg (*P*<.001).[e]
	Hurkmans 2018 [52]	Scale not specified	• Significantly more participants in 3 intervention groups lost at least 5% of their body weight compared with the control group.<br>• More participants in the combined group lost 5% or more compared with the app group (19%, *P*=.06).<br>• In the conventional group, app group, and combined group, BMI decreased significantly (*P*<.01, *P*<.01, and *P*<.001, respectively).
Fat mass reduction	Lee 2010 [23]	Bioelectrical impedance analysis with InBody 720	Reduction of fat mass in 1.2 kg (ss) (i) and 0.5 kg (ns) (c).
	Oh 2015 [33]	Bioelectrical impedance analysis with InBody U20	Statistically significant reduction, *P*=.001.
	Lee 2016 [43]	Bioelectrical impedance analysis with InBody 720	Before: 31.34%, after 30.87% (ns).
Waist circumference reduction	Block 2015 [28]	No information	Mean reduction intervention group 4.56 (95% CI −4.69 to −4.43); control group 2.22 (95% CI −2.36 to −2.09) (*P*<.001).

	McCarroll 2015 [32]	Spring-loaded tape measure (Gulick Tape Measure, Perform Better)	Before: 108.1 (SD 14.9) cm; after intervention: 103.7 (SD 15.1) cm. (*P*<.001).
	Safran 2015 [37]	Measured on the navel	No changes between intervention and control group were measured.
	Hutchesson 2016 [41]	A 0.1 cm using a nonextensible steel tape measure	Reduction in waist circumference 0.7 (1.4) cm (*P*=.04).[e]
	Lee 2016 [43]	Bioelectrical impedance analysis with InBody 720	• Waist circumference: before: 33.5 cm, after 33.3 cm (*P*<.05).<br>• Waist-hip ratio: before: 0.91, after 0.90. (ns).
	Willey 2016 [46]	Physician's office	Reduction by 7.2 cm or 6.6% from baseline, *P*=.005.
	He 2017 [49]	Auto-reported measure	No significant decrease: control group 2.39 (3.91) and intervention group 2.74 (4.48) cm.[e]
	Hurkmans 2018 [52]	Inelastic tape perpendicularly to the long axis of the body while the subject stood balanced on feet.	Within the conventional group, app group, and combined group, a decrease in metabolic risk factors was found, but this change was not significant (*P*=.12, *P*=.15, and *P*=.23). It does not specify which other outcomes are considered together with waist circumference.
Hip circumference	Fukuoka 2015 [30]	Standard protocol (not specified)	The intervention group had greater reductions in hip circumference (*P*<.001).
Change in physical activities	Bond 2014 [26]	SenseWear Mini Armband monitor	Percent time spent in both light (*P*<.05) and moderate-to-vigorous (*P*<.01) PA[f] was significantly increased compared with baseline.

	Finkelstein 2015 [29]	Step count measured via Fitbit	• Higher average daily number of steps in the intervention group (ns).<br>• Inactivity lower in the intervention group (25%) compared with the control group (30%) (*P*<.02).
	Fukuoka 2015 [30]	Omron Active Style Pro HJA-350IT pedometer	Intervention participants increased their daily step count by a mean of 2551 (4712) steps (a 38% increase) compared with a mean decrease of 734 (3308) steps (an 11% decrease) among controls (*P*=.02).
	Martin 2015 [31]	Data tracking by accelerometer	Control participants attained a mean of 616 fewer steps/day (6% decrease). Intervention participants increased their steps/day by a mean of 408 (4% increase).
	McCarroll 2015 [32]	Logs from the app health care provider interface	PA increased from 77.5185 (156.6) kcal expended and 22.7 (44.0) min to 1971.8 (1105.4) kcal and 182.3 (196.6) min (*P*=.001).[e]
	Oh 2015 [33]	IPAQ[g]-questionnaire [64]<br>MET tracking	No significant differences.
	Partridge 2015, 2016 [34,35]	IPAQ-SF[h] [65]	Number of PA days increased more in the intervention group (*P*=.003) compared with the control group (*P*=.02).
	Safran 2015 [37]	Questionnaire based on IPAQ [64]	The mean change in the weekly duration of PA was increased by 63 (20.8) min in the app group and reduced by 30 (−27.5) min in the control group (*P*=.02).[e]

	Spook 2015 [38]	Ad hoc questionnaire	No differences between intervention and control groups.
	Svetkey 2015 [39]	Paffenbarger questionnaire [66]	No significative changes in PA performance (kcal/week) in any of the 3 groups: control, personal coach, or cellular phone interventions.
	Aschbrenner 2016 [40]	Cardiorespiratory fitness with the 6-MWT[i] [67]	• Clinically significant improvements in cardiovascular fitness defined as >50 m increase on the 6-MWT (17%).<br>• Overall change in fitness was not significant.
	Quintiliani 2016 [45]	IPAQ [64]	Moderate and vigorous PA increased 545 and 792, respectively, MET[j] minutes per week (ns).
	Garcia-Ortiz 2018 [48]	ActiGraph GT3X accelerometer<br>7-day PA Record<br>Semistructured interview where	• Decrease of PA in both groups<br>• The intervention subgroup with high app adherence had better behavior than the low adherence subgroup (ss).
	Hurkmans 2018 [52]	Triaxial accelerometer (ActiGraph wGT3X-BT)	• No significant group PA time effects found.
Changes in dietary pattern	Nollen 2014 [27]	24-hour standardized dietary record [68]	• Fruit and vegetable consumption increased (+0.9, *P*=.08).<br>• Sugar-sweetened beverages consumption decreased (−0.3, *P*=.09).
	McCarroll 2015 [32]	Dietary logs from the app health care provider interface.	No significant differences in the macronutrient categories.
	Fukuoka 2015 [30]	Block Food Frequency Questionnaire [61]	• Greater reduction in intake of saturated fat (*P*=.007) in the intervention group.

			• Greater reductions in intake of sugar-sweetened beverages in the intervention group (*P*=.002).
	Partridge 2015, 2016 [34,35]	Questionnaires [69,70]	• Fruit and vegetable intake: nonsignificant difference.<br>• Intervention participants more likely to consume greater quantities of vegetables (*P*=.009).<br>• Sugar-sweetened beverage: Intervention participants consumed less (*P*=.002).
	Safran 2015 [37]	The Diet Quality Questionnaire [71]	• App users improved their score significantly at the end of the study from 67 (9.8) to 71 (0.6) (*P*<.001). No changes seen in the control group.[e]<br>• Success score (represents the success in maintaining healthy lifestyle) was higher among the app group (68%) compared with 36% in the control group (*P*<.001).
	Svetkey 2015 [39]	Healthy Eating Index [72]	No significant changes in any of the groups.
	Oh 2015 [33]	Daily meal self-tracking	No significant changes in any of the groups (*P*=0.12).
	Spook 2015	Ad hoc questionnaire	No differences between intervention and control groups.
	Quintiliani 2016 [45]	PrimeScreen [73] and Beverage Questionnaires (BEVQ-15) [74]	• Daily fruit and vegetable servings increased (ns).<br>• Diet composition score increased by a mean of 6.8 (ss).

			• Fluid ounces of sugar-sweetened beverages mean increased.
	García-Ortiz 2018 [48]	Mediterranean Diet Adherence Screener [75]	Both groups (intervention and control) increased adherence to Mediterranean diet with no differences between them.
	Mummah 2017 [50]	Harvard FFQ[k] [76]	Daily vegetable consumption was significantly greater in the intervention versus control condition: 2.0 servings; *P*=.04 for FFQ.
	Hurkmans 2018 [52]	Digital FFQ [77]	All groups reduced their total energy intake; only significant changes were found within the 3 intervention groups: conventional group (*P*<.01), app group (*P*<.01), and combined group (*P*<.001) and not in the control group (*P*=.22).
Emotional well-being	Pretlow 2015 [36]	Likert scale	• Self-esteem improvement: 2.78 (0.19) baseline and 3.59 (0.17) program completion (*P*<.01).[e] • Less likely to turn to food when stressed: 1.93 (0.18) baseline and 3.22 (0.22) program completion (*P*<.01).[e]
	McCarroll 2015 [32]	Functional Assessment of Cancer-Therapy-General (FACT-G) [78] and Weight Efficacy Life-Style Questionnaire (WEL) [79]	No statistically significant differences in quality of life measures (*P*>.05).
Screen time	Nollen 2014 [27]	Questionnaire of television viewing and computer use	No significant associations were seen between the device utilization and screen time.

Biochemical measurements	Fukuoka 2015 [30]	Nondefined	No differences at 5 months post intervention of blood levels of fasting lipids or glucose between control and intervention group.
	Oh 2015 [33]	Nondefined	No differences between the 2 groups.
	Block 2015 [28]	Nondefined	The ratio of TG[l]/HDL[m] reduced in intervention group (mean −0.21, 95% CI −0.30 to −.012); it was increased in the control group (mean 0.21, 95% CI 0.12-0.29) (*P*=.04).
	Willey 2016 [46]	Nondefined	• HDL levels increased 4.0 mg/dL (*P*=.04) and trend toward a reduction in total cholesterol of 10.5 mg/dL (*P*=.07) and triglycerides of 27 mg/dL (*P*=.07).<br>• A slight and nonsignificant reduction of HbA_{1C}[n]; mean values reduced from 5.5 to 5.4%.
	Hurkmans 2018 [52]	CardioChek Point-of-Care Self-Test device<br>Glucose BGStar measurement (Sanofi)	Within the conventional group, app group, and combined group, a decrease in metabolic risk factors was found, but this change was not significant (*P*=.12, *P*=.15, and *P*=.23). No specific results for glucose or fasting lipids are shown.
Blood pressure	Fukuoka 2015 [30]	Standard protocol	The intervention group had greater reductions in blood pressure, both SBP[o] and DBP[p] (*P*=.005).
	Willey 2016 [46]	Nondefined	SBP and DBP were significantly lower. Mean SBP and DBP fell 18.6 and 6.4 mmHg (*P*<.01).

	Mao 2017 [51]	Change in SBP	Mean reduction in SBP after 4 months in the intervention group 6.0 (1.6) (*P*=.002).[e]

[a]ss: significant (*P* value not available).

[b]ns: nonsignificant (*P* value not available).

[c]ITT: intention to treat analysis.

[d]BMI: body mass index (measured as kg/m^2).

[e]Values are expressed as mean (SD).

[f]PA: physical activity.

[g]IPAQ: International Physical Activity Questionnaires.

[h]IPAQ—SF: International Physical Activity Questionnaires—Short Form.

[i]6-MWT[i]: 6 minute walking test

[j]MET: metabolic equivalent of task.

[k]FFQ: Food Frequency Questionnaire.

[l]TG: triglycerides.

[m]HDL: high-density lipoprotein level.

[n]HbA_{1c}: hemoglobin A_{1c}.

[o]SBP: systolic blood pressure.

[p]DBP: diastolic blood pressure.
